# Efficacy and safety of immune checkpoint inhibitors in metastatic penile squamous cell carcinoma: a retrospective multicenter analysis

**DOI:** 10.1186/s40164-025-00629-4

**Published:** 2025-03-12

**Authors:** Timothy Schieber, Kelly Brunk, Anna Clennon, Benjamin L. Woolbright, Leonidas E. Bantis, Dennis Grauer, Tiewei Cheng, Saqib Abbasi, Elizabeth Wulff-Burchfield, Rahul Parikh, Haoran Li

**Affiliations:** 1 The University of Kansas Cancer Center, 2650 Shawnee Mission Pkwy, Westwood, KS 66205 United States of America; 2 National Community Oncology Dispensing Association, 9 Albany St #2e, Cazenovia, NY 13035 United States of America; 3 Aurora St Luke’s Medical Center, 2900 W Oklahoma Ave, Milwaukee, WI 53215 United States of America; 4 Department of Biostatistics and Data Science, University of Kansas Medical Center, 3901 Rainbow Blvd Mailstop 1026, Kansas City, KS 66103 United States of America; 5 The University of Kansas School of Pharmacy, 2010 Becker Dr, Lawrence, KS 66047 United States of America

**Keywords:** Penile, Squamous cell carcinoma, Immunotherapy, Pembrolizumab, HPV, Metastatic

## Abstract

Penile squamous cell carcinoma (PSCC) is a rare malignancy, and first-line therapy typically involves cisplatin-based chemotherapy. However, these regimens are often unsuitable for patients with poor performance status or advanced age due to their toxicity. This retrospective, multicenter study evaluated patients with metastatic PSCC who received immune checkpoint inhibitor (ICI) therapy between 2017 and 2024. A total of 10 patients met the inclusion criteria. The median age was 75 years, and 50% had an ECOG performance status of 2 or higher. The objective response rate (ORR) was 30%, and the disease control rate (DCR) was 40%. The duration of response and disease control was over 12 months and 8 months respectively in all patients with response or disease control. The median progression-free survival (PFS) was 2.82 months, and the median overall survival (OS) was 4.32 months. PD-L1 and HPV-positive patients had a 67% response rate (*n* = 2/3). No patients experienced severe immune-related adverse events (irAEs). This multicenter retrospective analysis suggests that ICI monotherapy may be a promising treatment option for patients with advanced PSCC who are either ineligible for first-line platinum-based chemotherapy or who have progressed after platinum-based chemotherapy, including those with poor performance status. Further studies are needed to confirm these findings and identify baseline patient characteristics that may optimize selection criteria.

**Supplementary Information:**

The online version contains supplementary material available at 10.1186/s40164-025-00629-4.

To the editor,

Penile squamous cell carcinoma (PSCC) is a rare malignancy, with treatment recommendations for metastatic disease based on small, single-arm, nonrandomized trials [1]. Due to its low incidence, first-line treatment options for metastatic PSCC historically included cisplatin-based chemotherapy with paclitaxel, ifosfamide, and cisplatin (TIP), or cisplatin plus fluorouracil (5-FU) [2, 3]. Preliminary results from the HERCULES trial evaluating cisplatin or carboplatin, 5-FU, and pembrolizumab are promising, though objective response rates (ORR) remain similar to TIP and cisplatin plus 5-FU across nonrandomized trials, with significant toxicity noted in all regimens [2–4]. Immunotherapy monotherapy has shown varying degrees of efficacy, with the largest global cohort reporting a poor ORR of 13% (*n* = 11/85) in a heterogeneous, heavily pretreated population [5]. 

This study presents a retrospective, multicenter analysis of immune checkpoint inhibitors (ICIs) for the treatment of metastatic PSCC. Patients were included if they received an ICI as monotherapy or in combination with other therapies, with the primary endpoint being ORR. No funding was used.

A total of 10 patients were identified. Key demographics included a median age of 75 years (range, 50–92) and 5 patients (50%) with an ECOG performance status (PS) of 2 or higher (Table 1). The ORR was 30%, with one complete response. Duration of response exceeded 12 months in all responding patients, with two patients showing ongoing responses at the data cut-off (Fig. 1). The disease control rate (DCR) was 40%, with one patient experiencing stable disease and a progression-free survival (PFS) of 8.3 months. The median PFS and overall survival (OS) were 2.82 months (range, 1.03–14.33) and 4.32 months (range, 1.03–24.93), respectively (Fig. 1). Among the 4 patients with disease control, 50% (n = 2/4) had an ECOG PS of 2 and were ineligible for cisplatin-based chemotherapy, receiving pembrolizumab as first-line therapy. The remaining 2 patients with disease control progressed on cisplatin-based chemotherapy and received pembrolizumab as second-line therapy. Three patients had p16 and PD-L1 positive disease, with a 67% (n = 2/3) ORR for both characteristics. The ORR and DCR for pembrolizumab monotherapy were 50% (n = 3/6) and 67% (n = 4/6), respectively. No other immunotherapy regimens showed disease control in this cohort, and no patients developed grade 3 + immune-related adverse events (irAEs). 

**Fig. 1:**
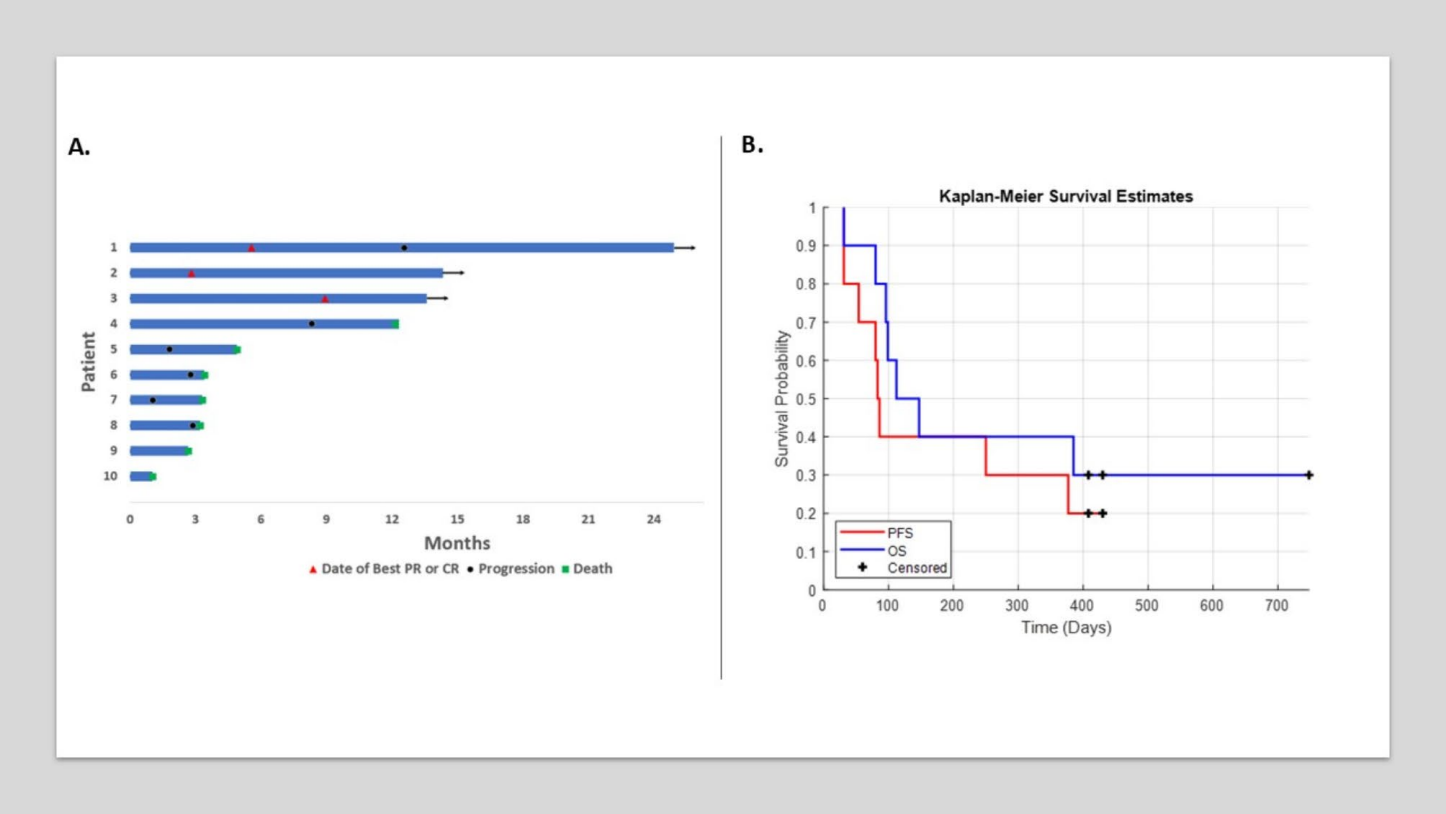
Efficacy outcomes

**Table 1:** Patient demographics, safety, and efficacy

Variable	*N* = 10
Age	
Median (range)	75 (50–92)
ECOG PS	
1 2 3	5 4 1
Race	
Caucasian Hispanic	9 1
BMI	
Median (range)	25.6 (19.6–46.3)
p16 Status	
Not Available Negative Positive	3 4 3
PD-L1	
Not Available Negative Positive (Values)	4 3 3 (84%, 5%, 30%)
MSI-H or dMMR	
Not Available Stable	4 6
Tumor Mutational Burden (TMB)	
Not Available TMB > 10 mutations/MB (Value) TMB < 10 mutations/MB (Value)	4 1 (12.43) 5 (9, 6.3, 6, 5, 4)
Surgical History	
None Radical or Partial Penectomy Inguinal lymph adenectomy	3 6 1
Radiation History	
Yes No	5 5
Line of therapy	
1 2 3+	3 5 2
Immunotherapy Regimen	
Cemiplimab Pembrolizumab Nivolumab Carboplatin + paclitaxel +pembrolizumab	1 6 2 1
Objective Response Rate (%)	
Complete response Partial response Stable disease Progressive disease	3 (30%) 1 (10%) 2 (20%) 1 (10%) 6 (60%)
Progression Free Survival (months)	
Mean (STD) Median (range)	6.10 (5.2) 2.82 (1.03–14.33)
Overall Survival (months)	
Mean (STD) Median (range)	8.45 (7.3) 4.32 (1.03–24.93)
12-month Progression Free Survival rate	3 (30%)
12-month Overall Survival Rate	4 (40%)
Any Grade irAE	
• Fatigue • Arthralgia • Rash • Hypothyroidism	4 (40%) 2 (20%) 2 (20%) 1 (10%)
Therapy Interruption due to any AE	0 (0%)
Grade 3 + irAE	0 (0%)
Discontinuation due to irAE	0 (0%)

In this multicenter retrospective analysis, clinically meaningful response rates with durable disease control were observed with immunotherapy in both the first-line and subsequent lines of therapy for metastatic PSCC. Platinum-based chemotherapy regimens such as TIP, cisplatin plus 5-FU, or platinum plus 5-FU plus pembrolizumab yield response rates ranging from 25 to 50%, but no comparative evidence exists [2–4]. These regimens have established efficacy, but cisplatin eligibility and tolerability remain challenges as shown by 51.4% of patients experienced grade 3 or higher adverse effects in the HERCULES trial [2–4]. In our cohort, 50% (*n* = 5/10) had an ECOG PS of 2 or higher, and the median age was 75, making many patients ineligible for these regimens due to tolerability concerns. The ORR of 30%, with responses lasting over 12 months in all responding patients and no grade 3 + irAEs, provides evidence that immunotherapy may offer a safe and effective alternative in the first-line setting.

For patients who have progressed on first-line platinum-based chemotherapy, treatment options are even more limited. Single-agent cetuximab and paclitaxel yield ORRs of approximately 20%, with OS durations of 29.6 weeks and 23 weeks, respectively [6, 7]. Pembrolizumab monotherapy was previously only available for microsatellite instability-high, mismatch repair-deficient, or tumor mutational burden-high tumors, based on prior basket trials limiting its access [8, 9]. The similar response rates in our cohort, along with numerically longer durations of response, suggest that immunotherapy in subsequent lines may be a high-quality option for patients with immunotherapy-naive metastatic PSCC.

Large observational studies on prognostic indicators for immunotherapy in PSCC are limited, though HPV and PD-L1 positive disease have shown improved outcomes in other cancers [10, 11]. This cohort contributes additional data, showing high response rates in HPV or PD-L1-positive disease. This cohort also highlights the tolerability of ICIs seen in similar cohorts [12].

In conclusion, this multicenter study demonstrates that immunotherapy leads to clinically significant response rates in metastatic PSCC, particularly for patients with poor performance status who are ineligible for cisplatin-based regimens in the first-line setting or after progression on platinum-based therapy. Additionally, it provides evidence of high response rates in PD-L1 and HPV-positive PSCC tumors.

## Electronic supplementary material

Below is the link to the electronic supplementary material.

Supplementary Material 1

## Data Availability

Data is provided within the manuscript or supplementary information files.
